# Different Types of Hyperfluorescence Observed in Post Anti-VEGF Fluorescein Angiographic Patterns in Retinopathy of Prematurity Patients

**DOI:** 10.3389/fmed.2021.800821

**Published:** 2022-01-24

**Authors:** Enzhong Jin, Hong Yin, Kailin Liu, Zhiqiao Liang, Mingwei Zhao

**Affiliations:** ^1^Department of Ophthalmology and Clinical Center of Optometry, Peking University People's Hospital, Beijing, China; ^2^Eye Diseases and Optometry Institute, Peking University People's Hospital, Beijing, China; ^3^Beijing Key Laboratory of Diagnosis and Therapy of Retinal and Choroid Diseases, Peking University People's Hospital, Beijing, China; ^4^College of Optometry, Peking University Health Science Center, Beijing, China

**Keywords:** fluorescein angiography, hyperfluorescence, retinopathy of prematurity (ROP), anti-VEGF (vascular endothelial growth factor) agents, treatment

## Abstract

**Purpose:**

To demonstrate that the demographic and treatment characteristics of retinopathy of prematurity (ROP) eyes showed different types of hyperfluorescence in fluorescein angiography (FA) initially treated with anti-vascular endothelial growth factor (VEGF) agents.

**Methods:**

A consecutive case series of ROP treated with anti-VEGF agents was retrospectively studied. All the patients underwent FA examinations at least 6 months later after treatment. The demographic and treatment characteristics of eyes with or without hyperfluorescence in FA were analyzed. The different types of hyperfluorescence were divided into three groups, including vascular leakage, fibrous membrane, and vascular abnormality.

**Results:**

Two hundred and forty-two eyes of 123 patients with treatment-required ROP were included. Hyperfluorescence was defined in 51/242 eyes, and 2.08 ± 1.11 injections were performed for them, while the eyes without hyperfluorescence received 1.65 ± 0.80 injections (*P* = 0.013). Vascular leakage was defined in 26/51 hyperfluorescence eyes. The postmenstrual age (PMA) of first injection for the hyperfluorescence group was 38.56 ± 3.24 weeks, which is earlier than that of infants without hyperfluorescence (*P* = 0.011). More additional treatments were performed in eyes with hyperfluorescence (23.53 vs. 3.66%, *P* = 0.000). Among them, the eyes with vascular leakage required more additional treatment than eyes without vascular leakage (42.31 vs. 4.00%, *P* = 0.004). For the 26 eyes with vascular leakage, 11 eyes of 8 patients received further treatments during further follow-up. No significant difference of refractive errors can be defined between different groups.

**Conclusion:**

Eyes with persistent hyperfluorescencein FA after treatment required more anti-VEGF and additional treatments, including laser and PPV. Not all hyperfluorescences were vascular leakage and required additional treatment.

## Introduction

Retinopathy of prematurity is a vasoproliferative disorder of immature retina developed in premature infants of low birth weight (BW) and young gestational age (GA) (1). Despite several treatment strategies developed in the past several decades, ROP remains a leading cause of childhood blindness worldwide (1).

The dysregulation of vascular endothelial growth factor (VEGF) associated with hypoxia has been identified as one of the major factors in the development of ROP and thought to play an important role in its abnormal vasculogenesis and neovascularization (2). According to this, anti-VEGF agents have been widely applied as an off-label therapeutic option for ROP as well as laser photocoagulation in the past decade (3–5). In recent years, the RAINBOW study has verified the superiority of 0.2-mg ranibizumab to laser therapy (6). According to the data of RAINBOW study (NCT02375971), ranibizumab has been approved as a new therapy for ROP in the European Union (EU), which is a milestone event for the application of the anti-VEGF agent in ROP.

Until now, the examination of binocular indirect ophthalmoscopy has been important for treatment decision, and whether a patient with ROP requires treatment or re-treatment is according to the recommendation of the Early Treatment for Retinopathy of Prematurity Cooperative Group (7). Fluorescein angiography (FA), as an imaging modality, which illustrates the vascular structure and provides more useful information for the observation of peripheral retinal vascular, can be an important adjunct to indirect ophthalmoscopy (8–10). In the past decade, peripheral vascular abnormalities, including incomplete vascularization, hyperfluorescent lesions, vascular leakage, vascular dilation, vascular blunting, and capillary dropout, have been reported in ROP (10–14).

In FA studies for ROP, hyperfluorescent lesions, including perivascular dye leakage, popcorn-like vascular abnormalities, focal capillary dilatation, coupled with capillary tuft formations, cotton wool, and rosary bead-like arterioles, have been reported before (8, 13). One of the most important hyperfluorescence findings observed in fluorescein angiogram can be vascular leakage located at the junction between vascular and avascular retina (13), which is an important indicator reflecting the peripheral vascular abnormalities and peripheral avascularity. It is not always easy to distinguish the vascular leakage from other hyperfluorescent lesions, which may affect the judgement of vascular development and treatment requirement.

In the present study, we tried to collect and analysis of the hyperfluorescence observed in angiograms of patients with ROP initially treated with VEGF inhibitors. The classification of hyperfluorescence based on the existence of leakage was first identified according to the FA outcomes at postmenstrual age (PMA) of approximately 25 months in our study. Different types of hyperfluorescence were illustrated and evaluated for the baseline characteristics and treatment requirements. It can provide valuable information for the management of ROP, especially for the eyes presenting hyperfluorescence in FA.

## Methods

### Study Design

This retrospective, institution-based cohort study of patients with ROP initially treated with VEGF inhibitors was approved by the Ethical Review Committee of Peking University People's Hospital (Beijing, China), which was conducted in accordance with the Declaration of Helsinki. Written informed consent was obtained from the parents of each infant before receiving the intravitreal injection of anti-VEGF agents and undergoing FA examination.

We retrospectively reviewed medical records and FA images of all included ROP patients that visited Children's Eye Center of Peking University affiliated to People's Hospital in Beijing, China between September 2014 and Jan 2020. FA images that showed hyperfluorescece were further analyzed.

### Patients

Two hundred and forty-two eyes of 123 patients with treatment-required ROP were finally included in this retrospective cohort study. All included patients were indicated for anti-VEGF treatment and were treated with conbercept and/or ranibizumab. All the eyes included fulfilled the criteria of treatment according to the guidelines of the Early Treatment for Retinopathy of Prematurity Cooperative Group (7). Only the patients with a follow-up period longer than 6 months were included. For all enrolled patients, the RetCam (Clarity Medical Systems, Inc., Pleasanton, CA) fundus color photographs and fluorescein angiograms were collected, as well as the medical chart.

### Treatment

Intravitreal injections of conbercept or ranibizumab were initially applied for patients with ROP in the present study. The eyes without regression of plus disease or ridge with one injection had a repeat injection during the follow-up period, as well as those eyes with recurrence of ROP after regression. The recurrence of ROP was defined as the recurrence of retinal abnormality such as ridge and plus disease after the regression of them (3). The laser photocoagulation, pars plana vitrectomy (PPV), and scleral buckling surgery were performed if necessary. In our center, the dose of both conbercept and ranibizumab for the infants with ROP was reduced to 0.25 mg/0.025 ml as half of the adult dosage for other retinal vascular diseases according to our previous study (3). The procedure of intravitreal injection was performed by an experienced retina specialist (H.Y.) according to the Royal College of Ophthalmologists ROP guideline of 2009 (8). The location of injections was in the temporal quadrant and 1.-1.5 mm posterior to the limbus. PPV surgeries were also performed by the same experienced retina specialist (HY).

### Fluorescein Angiography

Fluorescein angiographies were obtained under general anesthesia, as well as fundus photographs for all patients included. All examinations were performed by two experienced ophthalmologists (HY and EZJ). A 10% solution of fluorescein was injected intravenously with a dose of 0.1 ml/kg, followed by an isotonic saline flush. The FA examination lasted at least 5 min for each infant in our center. The FA digital images were blindly reviewed by three experienced ophthalmologists (HY, MWZ, and EZJ) without demographic information, and the recorded data included peripheral avascular zone, hyperfluorescence, circumferential vessels, and abnormal vascular branching. The avascular zone in the present study was defined and calculated as a distance from the ora serrata to the vascular terminus, with the unit of the diameter of papillary diameter (PD).

### Hyperfluorescence

The hyperfluorescence was collected from FA images of each patient by HY and EJ and divided into three types according to the characteristics of the angiograms. Three different types of hyperfluorescence, including vascular leakage, fibrous membrane, and vascular abnormality, were identified. For the further analysis of the association between the hyperfluorescence and treatment characteristics, the eyes with fibrous membrane and vascular abnormality were all assigned to the hyperfluorescence group with no vascular leakage.

### Statistical Analysis

The spreadsheet and statistical software (Microsoft Excel and SPSS 19.0; IBM, Armonk, NY) were used for statistical analyses. The independent sample *t*-test and chi-square test were used for the data comparison of two groups, and a *P* value less than 0.05 was considered as statistically significant. No other special test or analysis was used in this study.

## Results

Two hundred and forty-two eyes were finally included in the present study. Among the 242 eyes, 17 eyes were diagnosed with AP-ROP, while the other 225 type 1 ROPs with plus disease were also included (6 eyes with Zone I, Stage 2; 15 eyes with Zone I, Stage 3; 69 eyes with Zone II Stage 2; and 135 eyes with Zone II, Stage 3); all of them were initially treated with anti-VEGF agents. The average GA of the enrolled infants was 28.84 ± 2.43 weeks with a BW of 1173.91 ± 357.64 grams. Among them, 156 eyes have ever treated with conbercept and 142 eyes ever underwent intravitreal injection of ranibizumab. The first injection of anti-VEGF agent was performed at 39.76 ± 4.89 weeks PMA, while 123 of them required more than one treatment, and all included eyes received an average of 1.74 ± 0.89 injections. With a 20.83 ± 9.87-month follow-up, 19/242 eyes required additional treatment more than VEGF inhibitors to prevent the deterioration of disease. Among them, 8 eyes received PPV, and 12 eyes underwent laser photocoagulation. Ninety-two eyes were defined as myopia (SE < −0.25D) at the follow-up endpoint (Table 1).

**Table 1:** Demographic, clinical, and FA characteristics for included patients with ROP.

	**No. (%)**	**Mean ±SD**
Gender		
Male (*n*, %)	68 (55.28)	
Female (*n*, %)	55 (44.72)	
Diagnosis (*n*, eyes)		
Zone I, stage 2+	6 (2.48)	
Zone I, stage 3+	15 (6.20)	
Zone II, stage 2+	69 (28.51)	
Zone II, stage 3+	135 (55.79)	
AP-ROP	17 (7.02)	
Type of VEGF inhibitor		
Conbercept (*n*, %)	100 (41.32)	
Ranibizumab (*n*, %)	86 (35.54)	
Conbercept and Ranibizumab (*n*, %)	56 (23.14)	
Additional treatment except anti-VEGF (*n*, %)	19 (7.85)	
Laser photocoagulation (*n*, %)	12 (4.96)	
PPV (*n*, %)	8 (3.31)	
Hyperfluorescence (*n*, %)	51 (21.07)	
Vascular leakage (*n*, %)	26 (10.74)	
Fibrous membrane (*n*, %)	4 (1.65)	
Vascular abnormality (*n*, %)	21 (8.68)	
Circumferential vessels (*n*, %)	51 (21.07)	
Abnormal vascular branching (*n*, %)	106 (43.80)	
Myopia	92 (38.01)	
GA (weeks), mean ± SD		28.84 ± 2.43
BW (grams), mean ± SD		1173.91 ± 357.64
PMA at first injection (weeks), mean ± SD		39.76 ±4.89
Number of total injections, mean ± SD		1.74 ±0.89
PMA at FA (months), mean ± SD		25.32 ±9.52
Length of follow-up (months), mean ± SD		20.83 ±9.87
Avascular zone (PD), mean ± SD		2.31 ±1.20

*AP-ROP, aggressive posterior retinopathy of prematurity; PPV, pars plana vitrectomy; GA, gestational age; BW, birth weight; VEGF, vascular endothelial growth factor; PMA, postmenstrual age; FA, fluorescein angiography*.

In our series, hyperfluorescence can be defined in 51/242 eyes, and all observed hyperfluorescence in digital images was extracted and recorded. Among the eyes with hyperfluorescence observed in the FA images, 2.08 ± 1.11 injections were performed, while the eyes without hyperfluorescence received 1.65 ± 0.80 injections (*P* = 0.013). The PMA at first injection was 38.56 ± 3.24 weeks and 40.08 ± 5.20 weeks for eyes with and without hyperfluorescence (*P* = 0.011), while the mean avascular zone of them was 3.63 ± 0.85 PD and 2.63 ± 0.97 PD (*p* < 0.001). Significant more additional treatment except anti-VEGF can be observed in eyes with hyperfluorescence of 12/51 than eyes without hyperfluorescence of 7/191 (*P* = 0.000). Additionally, more eyes with abnormal vascular branching can also be observed in eyes with hyperfluorescence (68.63 vs. 37.17%) (*p* < 0.001). Other characteristics of FA images besides hyperfluorescence were also collected; circumferential vessels can be observed in 51/242 eyes, while abnormal vascular branching can be found in 106/242 eyes. Besides, myopia can be defined in 71/191 eyes without hyperfluorescence and 11/51 eyes with hyperfluorescence at the follow-up endpoint (*P* = 0.601) (Table 2).

**Table 2:** Demographic and treatment characteristics for patients with ROP with or without different types of hyperfluorescence.

	**No hyperfluorescence**	**Hyperfluorescence**			**P^a^**	**P^b^**
		**Total**	**Vascular leakage**	**No vascular leakage**		
Eyes, *n*	191	51	26	25	-	-
Males (*n*, %)	106 (55.50)	28 (54.90)	13 (50.00)	15 (60.00)	0.939	0.473
GA (weeks), mean ± SD	28.94 ± 2.45	28.48 ± 2.31	28.93 ±2.69	28.02 ± 1.75	0.231	0.158
BW (grams), mean ± SD	1176.54 ± 351.70	1164.02 ±379.20	1163.08 ±405.61	1165.00 ± 358.04	0.824	0.986
PMA at first injection (weeks), mean ± SD	40.08 ±5.20	38.56 ±3.24	38.90 ± 3.79	38.20 ± 2.57	0.011^*^	0.445
Total anti-VEGF injections, mean ± SD	1.65 ± 0.80	2.08 ±1.11	2.31 ±1.19	1.84 ± 0.99	0.013^*^	0.134
Avascular zone (PD), mean ± SD	2.63 ± 0.97	3.63 ±0.85	3.88 ±0.86	3.36 ± 0.76	0.000^**^	0.026^*^
Refractive errors, SE (D), mean ± SD	−0.13 ± 2.47	−0.33 ± 2.86	−0.69 ±3.44	0.04 ± 2.11	0.618	0.366
Myopia (*n*, %)	71 (37.17)	21 (41.18)	11 (42.31)	10 (40.00)	0.601	0.867
AP-ROP (*n*, %)	12 (6.28)	5 (9.80)	3 (11.54)	2 (8.00)	0.382	1.000
Zone I (*n*, %)	16 (8.38)	5 (9.80)	4 (15.38)	1 (4.00)	0.748	0.370
Zone II (*n*, %)	163 (85.34)	41 (80.40)	19 (73.08)	22 (88.00)	0.388	0.323
Additional treatment except anti-VEGF (*n*, %)	7 (3.66)	12 (23.53)	11 (42.31)	1 (4.00)	0.000^**^	0.004^**^
Circumferential vessels (*n*, %)	40 (20.94)	11 (21.57)	2 (7.69)	9 (36.00)	0.922	0.034^*^
Abnormal vascular branching (*n*, %)	71 (37.17)	35 (68.63)	17 (65.38)	18 (72.00)	0.000^**^	0.611

*GA, gestational age; BW, birth weight; PMA, postmenstrual age; AP-ROP, aggressive posterior retinopathy of prematurity; VEGF, vascular endothelial growth factor. P^a^, No hyperfluorescence vs. hyperfluorescence; P^b^, vascular leakage vs. no vascular leakage;*

* *p < 0.05*.

** *p < 0.01*.

**Figure 1:**
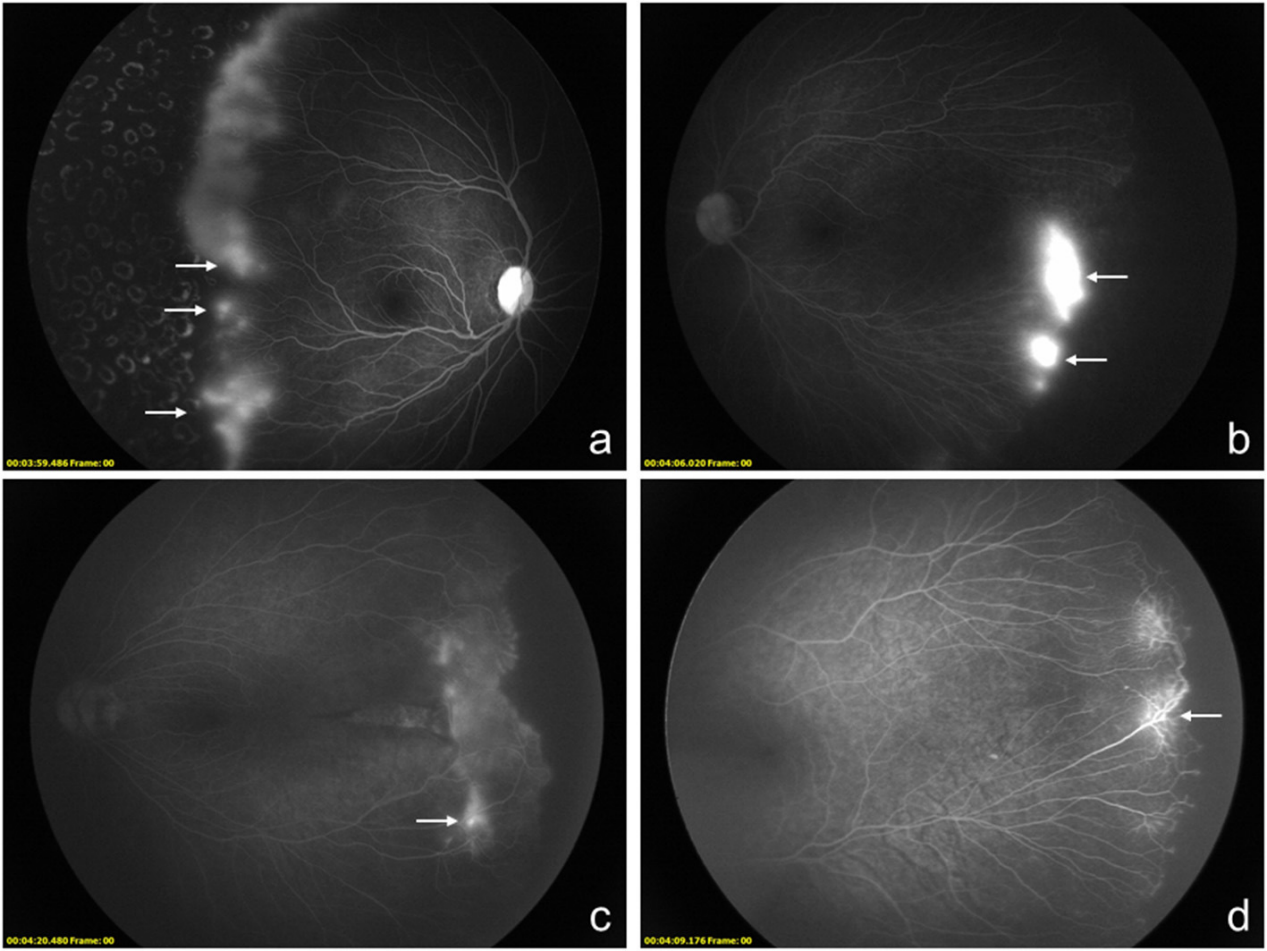
Montage of fluorescein angiography images of 4 different infants who received anti-VEGF agents as the primary treatment with the diagnosis of retinopathy of prematurity (ROP). **(a–d)**, Different types of hyperfluorescence lesions of the peripheral retina can be observed and categorized. The vascular leakage can be observed at **(a,b)**. The hyperfluorescence defined as fibrosis membrane is shown in **(c)**. As another kind of hyperfluorescence lesion, vascular abnormality is shown in **(d)**.

Different types of hyperfluorescence were analyzed and divided into three groups, including vascular leakage, fibrous membrane, and vascular abnormality, and the latter two groups were assigned to the hyperfluorescence group with no vascular leakage. Vascular leakage was defined in 26 eyes, while fibrous membrane and vascular abnormality were found in 4 and 21 eyes. In these three groups, the eyes with vascular leakage received 2.31 ± 1.19 injections, while those eyes with no vascular leakage received 1.84 ± 0.99 injections (*P* = 0.134). Besides a bigger avascular zone can be observed in eyes with vascular leakage (3.88 ± 0.86 vs. 3.36 ± 0.76, *P* = 0.026), more circumferential vessels can be defined in the group with no vascular leakage (36.00 vs. 7.69%, *P* = 0.034). No other demographic and treatment characteristics can be found between eyes with different types of hyperfluorescence (Table 2). Different types of hyperfluorescence and other characteristics of FA images are shown in Figure 1.

For the 51 eyes with hyperfluorescence in FA, further follow-up data were analyzed. With an 8.20 ± 7.08-month further follow-up, the eyes without leakage in FA received no additional treatment after the FA examination, although one of them underwent laser treatment when very young. Among the eyes with leakage, 11 eyes of 8 patients received further treatments, including anti-VEGF agents and laser. For this group, laser treatment was performed in 6 eyes of 5 patients after the discovery of leakage.

## Discussion

The hyperfluorescence observed in the FAs of ROP has been reported with different descriptions, including dye leakage, hyperfluorescent lesions, capillary malformations, and other vascular abnormalities (15). It is thought to play an important role in observation on pathogenesis and prognosis after treatment. Until now, no systematic and unified description of hyperfluorescence in FA has been reported. And, on the other hand, it is not always easy to distinguish the dye leakage from hyperfluorescent lesions or other vascular abnormalities, especially for the unskilled pediatric ophthalmologists. Therefore, much clear definition and classification of hyperfluorescence were required. The purpose of our present study is to discuss the hyperfluorescence observed in FA examinations for patients with ROP initially treated with anti-VEGF agents, and make comparison between different hyperfluorescence groups categorized by us for the demographic and treatment characteristics. We also report on the FA features observed after anti-VEGF treatment using definitions set by Lepore et al. (8, 13). The discussion of the categorized hyperfluorescence may attract more attention and provide detailed judgement for ophthalmologists to understand the fluorescein angiograms of ROP.

To the best of our knowledge, this is the largest retrospective cohort to show similar peripheral vascular abnormalities and hyperfluorescence in FA associated with using of anti-VEGF agents. Also, it is the first study that categorizes the hyperfluorescence into different types for the analysis of the demographic characteristics and relatively long-term treatment requirement for ROP.

The major findings in the current study included: First, hyperfluorescence existed in 51/242 eyes for ROP requiring anti-VEGF treatment, and those eyes underwent the first injection much earlier than eyes without hyperfluorescence. When the hyperfluorescence was divided into three types, only 26/51 (50.98%) eyes were defined as vascular leakage, while other 4 (7.84%) and 21 (41.18%) eyes were classified as fibrous membrane and vascular abnormality. Second, the eyes with performance of hyperfluorescence received significantly more anti-VEGF injections than the eyes with no hyperfluorescence in the FA images. Third, the eyes with hyperfluorescence had higher probability of additional treatment except anti-VEGF agents, 11/51 eyes underwent laser photocoagulation or PPV surgery, more than five times as much as eyes without hyperfluorescence (3.66%). A bigger avascular zone and higher proportion of abnormal vascular branching can be observed. Fourth, the eyes with hyperfluorescence defined as vascular leakage did not require more injections than the eyes with hyperfluorescence but no vascular dye leakage in the present cohort, but more additional treatment except anti-VEGF agents was required. Besides, a bigger avascular zone and lower proportion of circumferential vessels can also be defined in eyes with vascular leakage.

In our series, the hyperfluorescence only existed in almost one-fifth eyes requiring anti-VEGF treatment, which is much fewer than previous studies (10, 13, 14). In a recent study evaluating the FA findings of eyes primarily treated with ranibizumab, persistent vascular leakage has been evident in FA in 40% of eyes. And, in this study, 93.75% eyes received bilateral laser ablation treatment (14). In Lepore et al.'s study, different kinds of hyperfluorescent lesions were defined in eyes undergoing laser treatment, which also showed much more higher hyperfluorescence in FA (13). Since the scale of our study is much bigger and more infants only require anti-VEGF treatment were enrolled, fewer hyperfluorescence could be defined. On the other hand, the average PNA of FA examinations was 18.12 ± 9.29 months after birth for our study, which was also a bit later than the previous FA studies (10, 14).

Many previous studies have demonstrated promising outcomes of anti-VEGF treatment for ROP, and the recurrence of ROP was reported in 4–83% patients (3, 16, 17), which may be influenced by the types of ROP and other demographic characteristics. In our present study, the treatment frequency was calculated, and 1.74 ± 0.89 injections were performed for all included infants with 50.83% eyes required more than one injection. According to subgroup analysis, the patients in the hyperfluorescence group received an average of 2.08 ± 1.11 injections during the 20.83 ± 9.87-month follow-up period, which was significantly more than the no hyperfluorescence group of 1.65 ± 0.80 injections (*P* = 0.013). On the other hand, the eyes with hyperfluorescence received anti-VEGF treatment significantly earlier than eyes with no hyperfluorescence, which suggested the eyes with hyperfluorescence were much severe and required earlier treatment. But this point still required further investigation.

Although most ROP can achieve regression with monotherapy of anti-VEGF agents, additional treatments, including but not limited to laser photocoagulation, were required as necessary. In our cohort, 19/242 (7.85%) underwent additional treatment except anti-VEGF. For the hyperfluorescence group, 23.53% of eyes received additional laser photocoagulation or PPV surgery after primary anti-VEGF treatment, while only 3.66% patients of no hyperfluorescence group required additional treatment (*P* = 0.000). According to our data, the patients with hyperfluorescence required more anti-VEGF injections and more additional treatment. But, on the other hand, most FA examinations were performed later than 60-week PMA when the ROP was thought to be regression or stable with persistent vascular arrest (12). As we all know, the leakage was thought to require laser treatment traditionally (8). But, in this cohort, some eyes with vascular leakage receiving no laser treatment keep stable with no retinal detachment or disease progression until coming back for another FA examination. In 26 eyes defined with vascular leakage, only 11 eyes received further treatment, among which 6 eyes underwent laser photocoagulation. With a further follow-up period of 8.20 ± 7.08 months, 2 of them received a repeat treatment. For the other 15 eyes that did not accept additional treatment and under observation, no serious progression was observed until the end of the follow-up. It may suggest that not all hyperfluorescence or even leakage eyes must receive further treatment, but only close observation was required. Hyperfluorescence may be long-lasting in ROP eyes after anti-VEGF treatment. Besides, the mean avascular zone of the eyes with hyperfluorescence was bigger than that of eyes without hyperfluorescence, and the proportion of abnormal vascular branching can also be observed, both can be pieces of evidence of heavier vascular dysplasia for ROP eyes with hyperfluorescence.

Among the patients with hyperfluorescence in FA, nearly half of them showed vascular dye leakage, and the others were defined as fibrous membrane and vascular abnormality according to the continuous image of FA. Since the hyperfluorescence was divided into different types in our present study, the sub-group analysis was also performed. Among eyes with different types of hyperfluorescence, the eyes with vascular leakage showed lower proportion of circumferential vessels and a bigger avascular zone. The demographic and treatment data were collected and compared between eyes with or without vascular leakage; no significant difference of GA, BW, or treatment performance can be defined. As was concerned, the leakage may be stable as well as other kinds of hyperfluorescence like fibrous membrane and vascular abnormality, and did not require more further treatment than them. But further prospective cohort with a longer follow-up period is still required.

As a retrospective cohort, our study has several limitations. First, it was not prospectively designed and the enrolled patients received different VEGF inhibitors. But since both conbercept and ranibizumab were reported to be efficacy and safe for the treatment of ROP, the inclusion criteria would not affect the outcomes but can provide much comprehensive pieces of evidence (3–5). Second, the FA examinations were not observed dynamically, but only the digital images were reviewed. In order to improve the accuracy and reliability, all the images were reviewed by two ophthalmologists independently. Third, other structural and functional examinations as optical coherence tomography (OCT), perimetry, and visual acuity were not analyzed along with FA examinations in this study, which could better improve our understanding of ROP prognosis. Since most of the patients included in the present study were younger than 3 years old, these examinations cannot be well performed. As worth to be mentioned, the scale of the present study was relatively bigger than the previous studies, which can lead to a higher strength of conviction.

The results of this study confirm that there may be more than one-fifth ROP eyes showed hyperfluorescence even after primary treatment with VEGF inhibitors. Although more injections or more additional treatments, including laser photocoagulation and PPV, were required at the beginning for eyes with hyperfluorescence, no serious progression or retinal detachment can be observed nearly 1 year later after these treatments in the present study. Whether there is vascular leakage or not, the eyes with different types of hyperfluorescence did not show any significant difference of demographic characteristics.

Advances in imaging techniques such as the FA provide a new perspective for exploring the angiogenesis-related pathogenesis of ROP and also play an important role in understanding the treatment requirement and structural outcomes of ROP. The present study provides the primary evidence for ROP eyes with hyperfluorescence with or without vascular leakage that underwent anti-VEGF treatment initially. Eyes with persistent hyperfluorescence in FA after treatment usually required more anti-VEGF and additional treatments, including laser and PPV. But, according to our observation in this cohort, only half of the hyperfluorescences were vascular leakage and not all of them required additional treatment. Further prospective studies demonstrating the associated characteristics for ROP requiring further treatment or only need observation are still required.

## Data Availability Statement

The raw data supporting the conclusions of this article will be made available by the authors, without undue reservation.

## Ethics Statement

Written informed consent was obtained from the minor(s)' legal guardian/next of kin for the publication of any potentially identifiable images or data included in this article.

## Author Contributions

EJ: project development, data management, data analysis, manuscript writing, and editing. HY: project development, data management, manuscript writing, and editing. KL: data collection and manuscript editing. ZL: data collection, analysis, and manuscript editing. MZ: data management and manuscript editing. All authors contributed to the article and approved the submitted version.

## Funding

This study was supported by the National Key R&D Program of China (Grant No. 2020YFC2008200) and National Natural Science Foundation of China (Grant No. 81800850).

## Conflict of Interest

The authors declare that the research was conducted in the absence of any commercial or financial relationships that could be construed as a potential conflict of interest.

## Publisher's Note

All claims expressed in this article are solely those of the authors and do not necessarily represent those of their affiliated organizations, or those of the publisher, the editors and the reviewers. Any product that may be evaluated in this article, or claim that may be made by its manufacturer, is not guaranteed or endorsed by the publisher.
